# Mumps vaccine effectiveness of a 3rd dose of measles, mumps, rubella vaccine in school settings during a mumps outbreak -- Arkansas, 2016-2017

**DOI:** 10.1016/j.puhip.2023.100404

**Published:** 2023-06-30

**Authors:** Angela Guo, Jessica Leung, Tracy Ayers, Virgie S. Fields, Haytham Safi, Catherine Waters, Aaron T. Curns, Janell A. Routh, Dirk T. Haselow, Mariel A. Marlow, Mona Marin

**Affiliations:** a Division of Viral Diseases, Centers for Disease Control and Prevention, 1600 Clifton Road, Atlanta, GA, 30329, USA; b Epidemic Intelligence Service, Centers for Disease Control and Prevention, 1600 Clifton Road, Atlanta, GA, 30329, USA; c Arkansas Department of Health, 4815 W Markham St, Little Rock, AR, 72205, USA; d Council of State and Territorial Epidemiologists Applied Epidemiology Fellowship, 2635 Century Pkwy NE #700, Atlanta, GA, 30345, USA

**Keywords:** Outbreak control, Mumps, MMR vaccine, Vaccine effectiveness, Epidemiology

## Abstract

**Objectives:**

The largest mumps outbreak in the United States since 2006 occurred in Arkansas during the 2016-17 school year. An additional dose (third dose) of measles-mumps-rubella vaccine (MMR3) was offered to school children. We evaluated the vaccine effectiveness (VE) of MMR3 compared with two doses of MMR for preventing mumps among school-aged children during the outbreak.

**Study design:**

A generalized linear mixed effects model was used to estimate the incremental vaccine effectiveness (VE) of a third dose of MMR compared with two doses of MMR for preventing mumps.

**Methods:**

We obtained school enrollment, immunization status and mumps case status from school registries, Arkansas's immunization registry, and Arkansas's mumps surveillance system, respectively. We included students who previously received 2 doses of MMR in schools with ≥1 mumps case after the MMR3 clinic. We used a generalized linear mixed model to estimate VE of MMR3 compared with two doses of MMR.

**Results:**

Sixteen schools with 9272 students were included in the analysis. Incremental VE of MMR3 versus a two-dose MMR regimen was 52.7% (95% confidence interval [CI]: -3.6%‒78.4%) overall and in 8 schools with high mumps transmission it was 64.0% (95% CI: 1.2%‒86.9%). MMR3 VE was higher among middle compared with elementary school students (68.5% [95% CI: -30.2%‒92.4%] vs 37.6% [95% CI: -62.5%‒76.1%]); these differences were not statistically significant.

**Conclusion:**

Our findings suggest MMR3 provided additional protection from mumps compared with two MMR doses in elementary and middle school settings during a mumps outbreak.

## 1 Introduction

Mumps is an acute viral illness that typically presents as parotitis or other salivary gland swelling, but can also cause complications including orchitis, oophoritis, hearing loss, encephalitis, and meningitis. Mumps virus is transmitted person to person through direct contact or inhalation of respiratory droplets or saliva from an infected person [1]. To prevent mumps, the U.S. Advisory Committee of Immunization Practices (ACIP) recommends two doses of measles-mumps-rubella vaccine (MMR) routinely for children, with the first dose at age 12-15 months and a second dose at age 4-6 years, before school entry [2].

Before the introduction of mumps vaccine in the United States in 1967, mumps was a universal childhood disease, with >100,000 cases reported annually [3]. Following the implementation of a 1-MMR dose policy in the 1970s, and subsequent 2-MMR dose policy in the 1980s, mumps cases were reduced by >99%, with <300 cases reported each year in the early 2000s. Since 2006, there has been a resurgence of mumps cases mainly due to outbreaks in close-contact settings with high intensity exposure, like colleges or universities, and mainly affecting young adults who previously received two doses of MMR [4]. However, mumps outbreaks are also occurring in non-university settings and among school-aged children [3,5]. In 2017, ACIP recommended a third dose of MMR (MMR3) for persons who are identified by public health authorities as being at increased risk for mumps during mumps outbreaks to improve protection in outbreak settings [6]. However, gaps in our understanding of the impact of MMR3 still exist, especially in non-university settings. During the 2016-17 school year, the largest mumps outbreak in the United States since 2006 occurred in Northwest Arkansas. Most cases occurred among school-aged children with >90% of them having documentation of being up-to-date for mumps vaccination [7]. Consistent with the CDC guidance at the time, the Arkansas Department of Health (ADH) offered MMR3 to students in schools that had an attack rate ≥5 mumps cases per 1000 students. We evaluated the effectiveness of MMR3 for preventing mumps among school-aged children during the Arkansas mumps outbreak.

## 2 Methods

### 2.1 Setting

From August 5, 2016 to August 5, 2017, a large mumps outbreak with 2954 reported cases occurred in Northwest Arkansas. More than half (57%) of patients were members of the close-knit community that originated from the Marshall Islands, where large extended families often share living spaces and frequent social and religious gatherings are attended by large proportions of community members. Cases occurred in schools among Marshallese and non-Marshallese students, with 101 schools in 22 school districts reporting mumps cases during the outbreak period. Of all mumps cases associated with this outbreak, 58% (1676) occurred among school-aged children (aged 5–17 years), of whom 92% (1536) had previously been vaccinated with two doses of MMR [7].

Starting in August 2016, ADH recommended exclusion of unvaccinated or under vaccinated students until 26 days had passed without a new mumps case in the school. ADH also recommended an outbreak dose of MMR for all students in schools that had an attack rate ≥5 mumps cases per 1000 students and ongoing transmission. Unvaccinated or under vaccinated students who received a dose of MMR were allowed back into school immediately after vaccine receipt. Twenty-seven schools (16 elementary, 10 middle, and 1 high school) met the criteria for an outbreak dose of MMR. ADH conducted on-site MMR vaccination clinics in those schools (hereafter called intervention schools).

### 2.2 Case and immunization status ascertainment

#### 2.2.1 Mumps case identification

In Arkansas, as in all other US states, cases of mumps must be reported to the local public health authority. School nurses monitored school children for mumps and reported cases directly to the health department. The health department also instructed school nurses to follow up with students who were absent from school to monitor for mumps. Mumps cases were also reported to the health department by healthcare providers. Clinical and demographic data for all mumps cases reported to ADH were entered into Arkansas’ National Electronic Diseases Surveillance System (NEDSS).

Cases were classified as confirmed or probable using the 2010 Council of State and Territorial Epidemiologists mumps case definition [8]. A confirmed case was defined as an acute illness characterized by any of the following: acute parotitis or other salivary gland swelling lasting at least 2 days, aseptic meningitis, encephalitis, hearing loss, orchitis, oophoritis, mastitis, or pancreatitis, and the mumps virus detected via reverse-transcription polymerase chain reaction or culture (i.e., laboratory-confirmed). A probable case was defined as acute parotitis or other salivary gland swelling lasting at least 2 days or orchitis or oophoritis unexplained by another more likely diagnosis, and (1) a positive test for serum anti-mumps immunoglobulin M antibody, or (2) epidemiologic linkage to another probable or confirmed case or linkage to an affected group/community defined by public health during an outbreak of mumps.

School registries were used to identify students who attended intervention schools during the 2016-2017 school year. To identify which students had mumps, mumps cases that were reported in NEDSS to have occurred in an intervention school from August 16, 2016 to June 11, 2017 were cross-referenced with school registries. Matching was based on name, date of birth and sex of students; the majority (93%) of cases that were reported to have occurred in the intervention schools were successfully matched.

#### 2.2.2 MMR vaccination status

We used data from school registries to assess MMR vaccination status of students before the outbreak. The school registries indicated if a student had a complete MMR vaccination status in accordance with Arkansas school entry requirements (2 doses of MMR [9]). Data on MMR vaccination completion was provided by each school and was available for all students in the respective school. We considered that all students 6 years of age or older with a “complete vaccination” status documented in the school registries met Arkansas's school entry requirements and had received two doses of MMR. The Arkansas Department of Health does grant immunization exemptions for medical, religious or philosophical reasons [9]. Students who are exempted are noted with an “exempt” status and were not included in the analysis. Schools excluded unvaccinated students and staff until 26 days had passed without a new case [7]. MMR doses administered during the outbreak were recorded in the Arkansas Electronic Immunization System (WebIZ). WebIZ was used to ascertain receipt of MMR3 by identifying which students received a dose of MMR on the date of the MMR clinic at their school.

### 2.3 Analysis

Each school was treated as a separate unit of analysis given the intervention schools differed by the level of circulation of mumps (i.e., mumps attack rate), risk for mumps exposure, uptake of MMR3, and implementation of the MMR clinic relative to the peak of the outbreak. Attack rates and outbreak duration (interval from onset in first case until onset in last case) were calculated for each school. We used Fisher's exact test to compare differences in attack rates among students vaccinated with three or two MMR doses.

We used a generalized linear mixed effects model to estimate the incremental vaccine effectiveness (VE) of a third dose of MMR compared with two doses of MMR for preventing mumps. The model included mumps illness as the outcome and receipt of MMR3 as the exposure variable. We examined VE overall and stratified by the variables identified as associated with mumps illness in univariable analysis. To account for school-specific effects (e.g. age of students, uptake of MMR3, time between peak of cases and vaccination clinic), school was specified as random effect in all the models. VE was calculated as one minus the odds ratio times 100. To account for other variables that may influence a student's risk for mumps in this outbreak, we performed univariable logistic regression models to identify variables associated with mumps illness. We examined: school type (elementary or middle), time between peak of cases and vaccination clinic (<14 days or ≥14 days), and time between clinic and onset date of last case (<2 months or ≥2 months). Outbreak peak for each school was estimated using 5-day moving averages. Due to some schools having sparse events (mumps cases), as a proxy for ongoing mumps transmission, we performed subset analyses of only the schools with the highest attack rates (defined as higher than the median across schools among 2 dose MMR recipients).

The period included in the VE analysis (risk window) started 21 days after the vaccination clinic in each school to account for the time needed to mount an immune response to MMR3 and ended at the onset of the last mumps case in the respective school. We used a 21-day window after vaccination based on the mumps incubation period which ranges from 12 to 25 days, with an average of 16-18 days [3]. Additionally, we performed sensitivity analyses by estimating incremental VE using 7, 14, and 28-day post-MMR3 windows to account for the uncertainty in the time needed to mount an immune response.

We excluded from the VE analysis 1) students without a “complete vaccination” status or those younger than age 6 to ensure that only students vaccinated with 2-doses MMR were included in the analysis, 2) students who received a dose of MMR on dates of vaccination clinics at other schools or on dates of the five largest community vaccination clinics to avoid misclassification of third dose status, 3) students who became a mumps case before the start of the risk window because they would no longer be considered at risk for mumps, and 4) schools that did not have at least one mumps case in the risk window to ensure that we included schools in which mumps was circulating and exposure of students was occurring.

Data were analyzed using SAS software, version 9.4.

This project was submitted for human subjects evaluation at the Centers for Disease Control and Prevention and was determined to be non-research activity.

## 3 Results

### 3.1 Study population

**Fig. 1:**
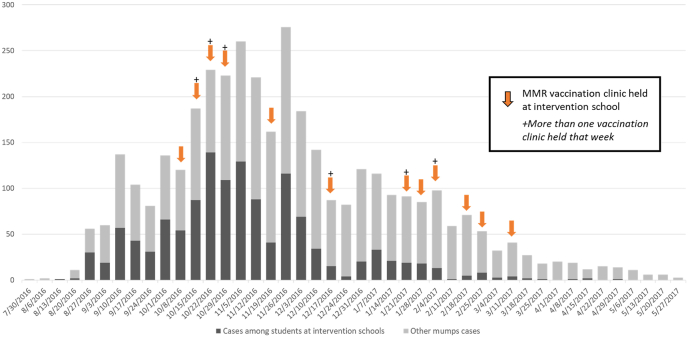
Mumps cases in Arkansas, USA, and in the 27 intervention schools by week of illness onset, and date of school MMR vaccination clinics, Aug 1, 2016, to June 11, 2017 Note: Schools with vaccination clinics held on after the week of Jan 21, 2017 did not meet the criteria for inclusion in the VE study except the one with a clinic the week of Feb 25, 2017.

**Fig. 2:**
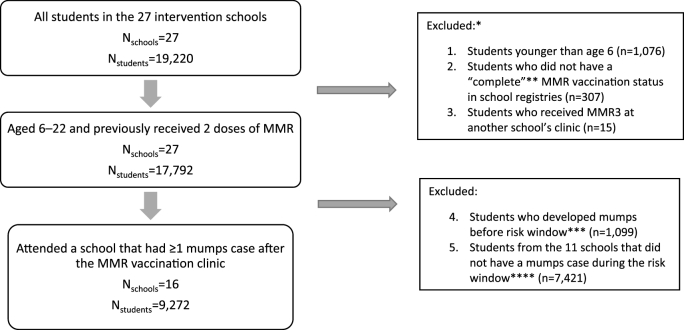
Flow diagram of study population.

A total of 19,220 students attended the 27 schools that held a MMR vaccination clinic during the study period (August 16, 2016 to June 11, 2017). Sixteen of these schools had at least one mumps case in the risk window following the MMR vaccination clinic. MMR vaccination clinics occurred at schools on different dates during the outbreak period (Fig. 1). The study population included 9272 students from these 16 intervention schools who had received 2-doses of MMR prior to the start of the outbreak (Fig. 2), 4687 students from 9 elementary schools and 4585 students from 7 middle schools (Table 1). The median size of the schools was 545 students (range: 422‒559) for the elementary schools and 628 students (range: 446‒935) for middle schools. MMR3 uptake at clinics varied by school (range: 2%‒33%); elementary schools tended to have higher MMR3 uptake (median: 23%, range: 3%‒33%) compared with middle schools (median: 9%, range: 2%‒18%). Other characteristics also varied by school and school type, with middle schools tending to have more mumps cases, longer lag times between the peak of mumps cases and the date of the MMR clinic, and longer mumps transmission post-vaccination clinics compared with elementary schools (Table 1).

**Table 1:** Characteristics of students and mumps transmission in the schools by school type, Arkansas, 2016-2017.

	Elementary (n = 9 schools, 4687 students)	Middle (n = 7 schools, 4585 students)	Total (n = 16 schools, 9272 students)
Students per school, median (range)	545 (422-559)	628 (446-935)	557 (422-935)
Student age, median (range)	8 (6-13)	13 (10-18)	11 (6-18)
Number of students who received MMR3 (no, %)	1053 (22%)	471 (10%)	1524 (16%)
MMR3 uptake per school, median (range)^a^	23% (3%-33%)	9% (2%-18%)	13% (2%-33%)
Number of mumps cases per school^a^, median (range)	51 (21-77)	66 (14-108)	51 (14-108)
Duration of outbreak (interval between onset dates of first and last case), in weeks, median^a^ (range)	20 (12-23)	21 (10-30)	20 (10-30)
Interval between peak cases and MMR clinic, in days, median (range)	15 (1-55)	23 (-12-37)^b^	20 (-12-55)
Interval between MMR clinic and onset date of last mumps case in school, in days, median (range)	45 (23-84)	99 (27-152)	56 (23-152)

MMR3 = Third dose of measles, mumps, rubella vaccine; MMR = Measles, mumps, rubella vaccine.

a Estimates based on entire outbreak period in each school. Includes students who developed mumps before the start of the mumps risk window following the MMR clinic in each school and who were excluded from the vaccine effectiveness analysis.

b Negative interval between peak cases and MMR clinic indicate situations in which the peak of the cases at that school occurred before the MMR clinic.

### 3.2 Risk for mumps

The final generalized linear mixed model included two covariates: receipt of MMR3 and school type. Among the entire study population, the attack rate among MMR3 recipients was significantly lower than the attack rate among two-dose recipients (4.6 vs 10.3 per 1000 population, p = 0.046) (Table 2). The difference in attack rates was greater among middle school students (4.2 vs 12.6 per 1000 population, p = 0.17) than elementary school students (4.7 vs 7.7 per 1000 population, p = 0.4). This pattern was consistent when the analysis was limited to schools with at least one mumps case reported ≥2 months following the MMR clinic or schools with the highest attack rates for two-dose MMR students. Mumps attack rates by individual school are provided in Suppl Table A.

**Table 2:** Mumps attack rates and incremental vaccine effectiveness among students at intervention schools when the risk window started 21 days after the MMR vaccination clinic, Arkansas, 2016-2017.

	Two MMR Doses			Three MMR Doses			p value^a^	Incremental vaccine effectiveness of MMR3 vs. 2 MMR doses, % (95% CI)^b^
	Mumps cases	Population at risk	Attack rate	Mumps cases	Population at risk	Attack rate		
	No. of students		No. of cases/1000 population	No. of students		No. of cases/1000 population		
All students	80	7748	10.3	7	1524	4.6	0.046	52.7 (-3.6, 78.4)
Elementary school	28	3634	7.7	5	1053	4.7	0.4	37.6 (-62.5, 76.1)
Middle school	52	4114	12.6	2	471	4.2	0.17	68.5 (-30.2, 92.4)
Schools (n = 8) with at least one mumps case ≥2months following the MMR vaccination clinic	60	4029	14.9	5	622	8.0	0.2	45.9 (-36.0, 78.5)
Schools (n = 8) with attack rates greater than median mumps attack rate for 2 doses MMR students^c^	67	4266	15.7	4	830	4.8	0.047	64.0 (1.2, 86.9)

MMR3 = Third dose of measles, mumps, rubella vaccine; MMR = Measles, mumps. rubella vaccine.

a Fishers exact test examining the difference in attack rates among 3 dose MMR and 2 dose MMR students.

b Incremental vaccine effectiveness of MMR3 compared with a two-dose regimen was estimated by using a generalized linear mixed which included mumps illness as the outcome and receipt of MMR3 as the exposure variable.

c Median attack rate = 12 per 1000 population.

### 3.3 Vaccine effectiveness

Using the final generalized linear mixed model, the incremental VE of a third dose of MMR versus a two-dose regimen for preventing mumps was estimated to be 52.7% (95% confidence interval [CI]: -3.6%‒78.4%) (Table 2). In univariable analysis, variables significantly associated with mumps illness were school type (elementary vs middle) and time between clinic and onset date of last mumps case (<2 months vs ≥2 months) (Suppl Table B). Therefore, the analysis was further stratified by these variables. Incremental VE was higher among middle schoolers compared with elementary school students (68.5% [95% CI: -30.2%‒92.4%] vs 37.6% [95% CI: -62.5%‒76.1%]) and was slightly lower (45.9% [95% CI: -36.0%‒78.5%]) among schools with at least one mumps case reported ≥2 months following vaccination clinic compared with the overall pooled estimate. These differences however were not statistically significant.

When the analysis was restricted to schools with the highest attack rates, incremental VE in these schools was slightly higher than the overall pooled estimate and statistically significant (64.0% [95% CI: 1.2%‒86.9%]). Considering other risk windows, the incremental VE ranged from 16.4% at 7 days post-vaccination to 63.5% at 28 days post-vaccination [Suppl Table C]. In each of these analyses, incremental VE of MMR3 versus a two-dose regimen was higher among middle school students than among elementary school students, though not statistically significant.

## 4 Discussion

Our findings suggest that a third dose of MMR provides additional protection from mumps compared to having only two MMR doses when given during a mumps outbreak in elementary and middle school settings. Despite relatively low MMR3 uptake, receiving MMR3 was associated with 52.7% reduction in the risk for mumps. These findings suggest that students can benefit from a third dose of MMR in school settings with ongoing mumps transmission due to an outbreak.

The point estimate of the incremental VE of MMR3 was higher among middle school students compared with elementary school students. This may suggest there is some waning of immunity after the second dose of MMR as time since receipt of last MMR would likely be longer for middle school students compared to elementary school students [11,12]. However, because dates of previous doses of MMR were not available, we were unable to examine time since the second MMR dose in this analysis. Cardemil et al. assessed time since last vaccination among college students during a university mumps outbreak and found that time since receipt of last MMR was associated with risk for mumps [10]. The findings in this study suggest that this waning may begin as early as middle school-age.

Even if the point estimate of the incremental VE indicates increased protection against mumps from MMR3, this finding was not statistically significant in our analysis. A small number of cases after the MMR vaccination clinics might have limited our ability to detect statistical significance. However, consistent indication of additional protection on all subanalyses suggest that the benefit of the third dose is real. Moreover, when the analysis was limited to situations with a higher likelihood for mumps transmission after the MMR3 clinics (i.e., schools with the highest mumps attack rate and at least 2 months of demonstrated mumps transmission post-MMR clinic), MMR3 appeared to be more protective.

We also conducted sensitivity analyses to account for the uncertainty in the time it takes to mount an immune response after MMR3. As expected, the incremental VE increased as the length of the window increased. We did not see any protective effect of MMR3 when using the 7-day window, which is consistent with the fact that MMR is not recommended as post-exposure prophylaxis for mumps [3]. For students who became mumps cases within 7 days of receiving a third dose of MMR, it is likely they were exposed and infected prior to vaccination, before they were able to mount a response from their MMR3 dose.

For this analysis, because mumps transmission and thus risk for mumps exposure varied by school, combining students from all schools would have led to an overall attack rate that would not reflect the real risk for disease among both the two-dose and the three-dose recipients. Using a random effects model addressed the variation among schools since each school was treated as its own unit of analysis. This method has also been used in the Ebola ring vaccination trials to assess VE in outbreak settings [13]. This analysis supports the appropriateness of a random effects model to estimate VE that can be used in other investigations where risk for disease and transmission vary by setting.

There were several limitations to this analysis. Small numbers of mumps cases and students who received MMR3 likely limited the ability to detect statistical significance of the impact of MMR3. Mumps transmission also occurred in the community, particularly in the Marshallese community therefore, some students may have had greater exposure to mumps than others, despite attending the same school. Data on students’ race/ethnicity were not available therefore, we could not control for this variable in the analysis. There was low uptake of MMR3 within schools, so it was difficult to evaluate if MMR3 vaccination can help reduce the size and duration of mumps outbreaks. Timing of clinics was affected by logistical challenges related to school holidays, particularly Thanksgiving and winter breaks. School holidays also might have affected the ability to detect mumps cases among students as we relied heavily on case reports from school nurses. Misclassification of vaccination status is possible given that previous receipt of 2 doses of MMR was inferred from records of “complete” MMR vaccination status and students might have received MMR3 at locations other than school during the outbreak. This included MMR clinics in the community, including in locations frequented by the members of the Marshallese community. Misclassification bias was minimized by restricting inclusion in the study of students aged ≥6 years for whom complete vaccination means having received two MMR doses, and by excluding students with a record of MMR on dates of vaccination clinics in other schools or in the community. Additionally, some students with a “complete” vaccination status may have received more than 2 doses before the outbreak.

These real-world findings further contribute to the evidence of a benefit from a third dose of MMR in settings where there is ongoing mumps transmission due to an outbreak. While there were several limitations that led to less precise VE estimates, the mumps attack rates were significantly lower among three-dose compared with two-dose MMR recipients, confirming previous findings from other large mumps outbreak studies [10,14]. MMR3 can provide individual protection against mumps even among school-aged children, particularly among middle school children where we saw a larger impact of MMR3 in reducing mumps risk. Immunological studies suggested the added protection from MMR3 may be short term, with boost in antibodies one month after vaccination that then declined to near-baseline levels after one year [15,16]. Additional studies in additional outbreak settings will better determine MMR3 VE and its impact in preventing outbreak-related mumps cases.

## Ethics approval

None sought.

The Arkansas Department of Health (ADH) collected the data for this analysis during the 2016-2017 mumps outbreak to coordinate response efforts, including identifying which schools needed vaccination clinics in accordance with Centers of Disease Control and Prevention (CDC) guidance at that time. ADH requested CDC assistance to respond to this outbreak in 2016 and shared data with CDC to support response efforts. ADH coordinated with the affected school districts and Arkansas Department of Education to receive approval to utilize school registry data for this analysis.

## Authors’ contributions

AG: data collection, study design, performed the analysis and wrote the manuscript. TA, JL: performed the analysis, participated in its design. JL, M. Marin, M. Marlow: study design and wrote the manuscript. AC, JR: study design. TA, DH, VF, HS, CW: data collection. All authors read and approved the final manuscript.

## Funding

This research did not receive any specific grant from funding agencies in the public, commercial, or not-for-profit sectors.

## Disclaimer

The findings and conclusions in this report are those of the authors and do not necessarily represent the official position of the Centers for Disease Control and Prevention.

## Declaration of competing interest

The authors declare that they have no known competing financial interests or personal relationships that could have appeared to influence the work reported in this paper.
