# Supplementary material for: Mumps vaccine effectiveness of a 3rd dose of measles, mumps, rubella vaccine in school settings during a mumps outbreak -- Arkansas, 2016-2017
Source: Public Health Pract (Oxf). 2023 Jun 30;6:100404. doi: 10.1016/j.puhip.2023.100404 (PMC10719407; doi:10.1016/j.puhip.2023.100404)
Supplement: Multimedia component 1 [file mmc1.docx]

**Supplement Tables/Figures:**

| Suppl Table A: Mumps attack rates by school | | | | | | | | | |
| --- | --- | --- | --- | --- | --- | --- | --- | --- | --- |
| School | 2 doses MMR | | | 3 doses of MMR | | | School Type | Two months or more between clinic date and onset date of last mumps case | 2 dose attack rate >median attack rate* |
|  | Mumps cases | Population at risk | Attack rate per 1000 students | Mumps cases | Population at risk | Attack rate per 1000 students |  |  |  |
|  | No. of students | |  | No. of students | |  |  |  |  |
| School A | 7 | 489 | 14.3 | 0 | 70 | 0.0 | Elementary |  | Y |
| School B | 4 | 314 | 12.7 | 1 | 108 | 9.3 | Elementary | Y | Y |
| School C | 4 | 360 | 11.1 | 1 | 195 | 5.1 | Elementary |  | Y |
| School D | 5 | 463 | 10.8 | 0 | 17 | 0.0 | Elementary | Y | Y |
| School E | 3 | 372 | 8.1 | 2 | 173 | 11.6 | Elementary | Y |  |
| School F | 2 | 482 | 4.1 | 0 | 77 | 0.0 | Elementary |  |  |
| School G | 1 | 313 | 3.2 | 0 | 162 | 0.0 | Elementary |  |  |
| School H | 1 | 401 | 2.5 | 0 | 139 | 0.0 | Elementary |  |  |
| School I | 1 | 440 | 2.3 | 1 | 112 | 8.9 | Elementary |  |  |
| School J | 14 | 526 | 26.6 | 2 | 102 | 19.6 | Middle | Y | Y |
| School K | 12 | 579 | 20.7 | 0 | 14 | 0.0 | Middle | Y | Y |
| School L | 11 | 645 | 17.1 | 0 | 92 | 0.0 | Middle | Y | Y |
| School M | 9 | 577 | 15.6 | 0 | 70 | 0.0 | Middle | Y | Y |
| School N | 2 | 553 | 3.6 | 0 | 46 | 0.0 | Middle | Y |  |
| School O | 3 | 871 | 3.4 | 0 | 64 | 0.0 | Middle |  |  |
| School P | 1 | 363 | 2.8 | 0 | 83 | 0.0 | Middle |  |  |
| Total | 80 | 7,748 | 10.3 | 7 | 1,524 | 4.6 |  |  |  |

*Median mumps attack rate for 2 doses MMR students = 12 per 1000

MMR = Measles, mumps rubella vaccine

| Suppl Table B. Odds of mumps* among students who received MMR3 (n=16 schools) | | |
| --- | --- | --- |
| Variable | Odds ratio (95% CI) | P-value |
| Received MMR3 |  |  |
| Yes | 0.44 (0.21, 0.96) | 0.04 |
|  |  |  |
| School type |  |  |
| Middle vs Elementary | 1.67 (1.08, 2.60) | 0.02 |
|  |  |  |
| Time between peak of mumps cases and MMR vaccination clinic |  |  |
| Less than 14 days | 1.31 (0.84, 2.02) | 0.23 |
|  |  |  |
| Time between MMR clinic and onset date of last case |  |  |
| 2 months or more | 2.94 (1.81, 4.76) | <0.0001 |
|  |  |  |
| *Univariable logistic regression models  MMR3 = Third dose of measles, mumps, rubella vaccine | |  |

| Suppl Table C: Mumps attack rates and incremental vaccine effectiveness among students at intervention schools when the risk window started 7, 14 or 28 days after the MMR vaccination clinic, Arkansas, 2016-2017. | | | | | | | | | | | | |
| --- | --- | --- | --- | --- | --- | --- | --- | --- | --- | --- | --- | --- |
|  | Two Doses MMR | | | | | | Three Doses MMR | | | p value | | Incremental vaccine effectiveness of MMR3 vs. MMR2 |
|  | Mumps cases | | Population at risk | | Attack rate | | Mumps cases | Population at risk | Attack rate |  | |  |
|  | no. of students | | | | Cases /1000 population | | no. of students | | Cases /1000 population |  | | % (95% CI) |
| Risk window started 7 days after the MMR vaccination clinic | | | | | | | | | | | | |
| All students | 143 | | 8,673 | | 16.5 | | 23 | 1,621 | 14.2 | 0.59 | | 16.4 (-31.6, 46.8) |
| Elementary | 52 | | 4,081 | | 12.7 | | 14 | 1,117 | 12.5 | 1 | | 10.5 (-63.8, 51.1) |
| Middle | 91 | | 4,592 | | 19.8 | | 9 | 504 | 17.9 | 0.87 | | 21.3 (-57.1, 60.5) |
| Risk window started 14 days after the MMR vaccination clinic | | | | | | | | | | | | |
| All students | | 121 | | 8,651 | | 14.0 | 13 | 1,611 | 8.1 | | 0.06 | 42.1 (-3.8, 67.7) |
| Elementary | | 42 | | 4,071 | | 10.3 | 8 | 1,111 | 7.2 | | 0.39 | 32.9 (-44.5, 68.8) |
| Middle | | 79 | | 4,580 | | 17.2 | 5 | 500 | 10.0 | | 0.27 | 49.9 (-24.3, 79.8) |
| Risk window started 28 days after the MMR vaccination clinic | | | | | | | | | | | | |
| All students | | 63 | | 6,551 | | 9.6 | 4 | 1,295 | 3.1 | | 0.02 | 63.5 (-1.4, 86.8) |
| Elementary | | 18 | | 3,312 | | 5.4 | 3 | 889 | 3.4 | | 0.6 | 37.9 (-110.9, 81.7) |
| Middle | | 45 | | 3,239 | | 13.9 | 1 | 406 | 2.5 | | 0.06 | 81.9 (-31.7, 97.5) |

MMR3 = Third dose of measles, mumps, rubella vaccine; MMR = Measles, mumps. rubella vaccine

MMR2 = 2 doses of MMR received before outbreak
